# Remote Cerebellar Haemorrhage: A Potential Iatrogenic Complication of Spinal Surgery

**DOI:** 10.1155/2018/5870584

**Published:** 2018-09-16

**Authors:** Muhammad Atif Naveed, Rajiv Mangla, Hajra Idrees, Rashi I. Mehta

**Affiliations:** ^1^Department of Radiology, Shaukat Khanum Memorial Cancer Hospital, Lahore, Pakistan; ^2^Department of Radiology, SUNY Upstate Medical University, Syracuse, NY, USA; ^3^Department of Radiology, West Virginia University, Morgantown, WV, USA

## Abstract

We report the case of a 51-year-old man with no significant past medical history, who underwent elective revision spinal surgery and subsequently developed intracranial hypotension, remote cerebellar haemorrhage (RCH), and mild hydrocephalus on the fourth postoperative day. Remote cerebellar haemorrhage is a known complication of supratentorial surgery. This iatrogenic phenomenon may also occur following spinal surgery, due to dural tearing and rapid cerebral spinal fluid (CSF) leakage, resulting in intracranial hypotension and cerebellar haemorrhage. This complication may result in severe permanent neurologic sequelae; hence, it is of pertinence to diagnose and manage it rapidly in order to optimise patient outcome.

## 1. Case Report

A 51-year-old male, with a remote previous history of L4-L5 spinal decompression and fusion, presented in our outpatient clinic with worsening lower back pain. Physical examination showed lumbar radiculopathy and neurogenic claudication, while a magnetic resonance imaging (MRI) scan of the lumbar spine revealed disc protrusions and high-grade spinal canal stenosis at the L2-L3 and L3-L4 levels. Consequently, he underwent elective spinal decompression revision surgery, with an extension of instrumented fusion from L2-L5.

**Figure 1:**
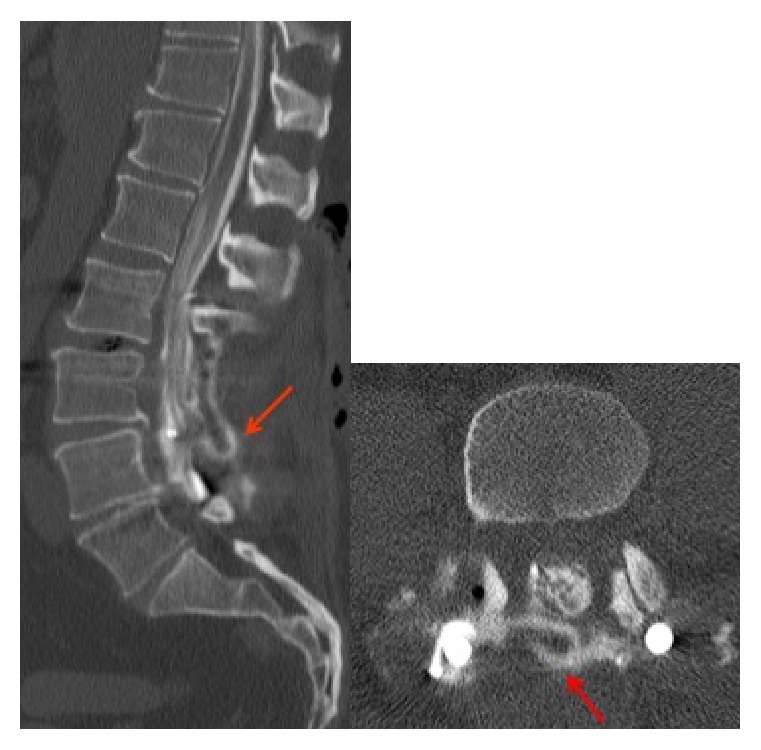
Sagittal and axial CT lumbar myelogram images show a focal CSF leak from the posterolateral aspect of the thecal sac at L3-L4 level, with iodinated contrast accumulating posterior to the thecal sac (red arrows).

On experiencing new onset persistent headaches on the second postoperative day, a computerized tomography (CT) myelogram was performed, and showed CSF leakage from a dural tear at the L3-L4 level (Figure 1). Soon after the CT myelogram, image-guided lumbar drain placement was performed, and 8cc of fibrin glue was injected at the site of the leak.

**Figure 2:**
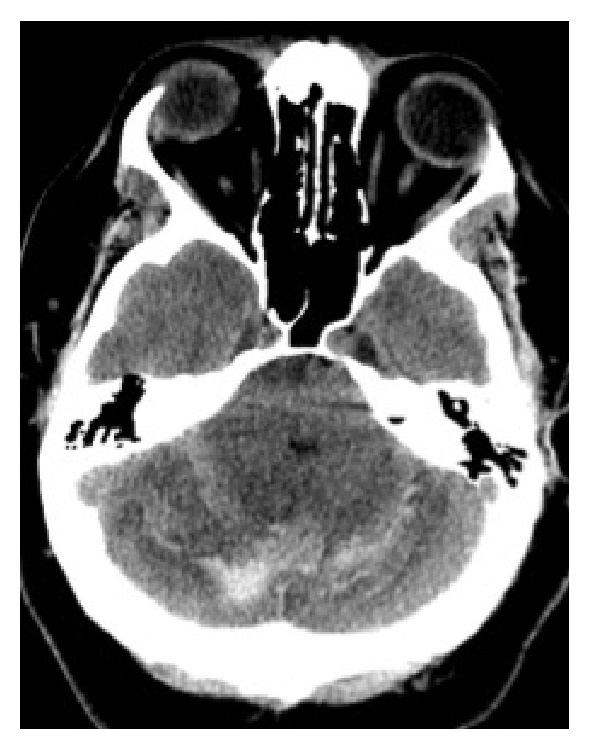
Nonenhanced CT head image at the level of the cerebellum demonstrates acute haemorrhages in both cerebellar hemispheres (arrow) with edema, causing mass effect on the fourth ventricle.

**Figure 3:**
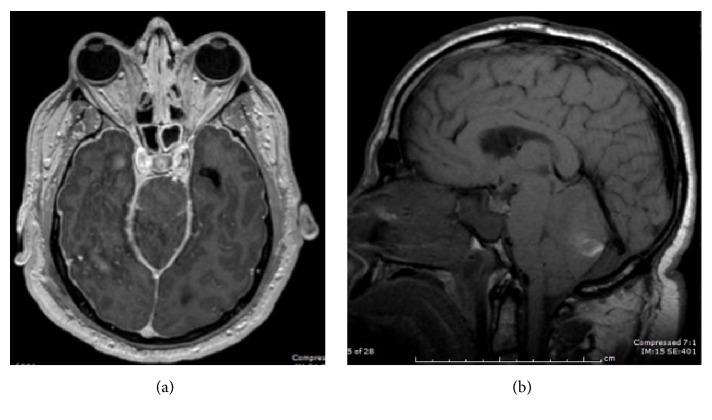
MRI brain was performed on the same date of the CT head. It confirmed cerebellar haemorrhage and mass effect on the 4th ventricle and brainstem. (a) Axial T1 SPGR postgadolinium MR image shows diffuse dural enhancement. (b) Sagittal T1 MR image of the brain shows “sagging” midbrain, flattening of the pons against the clivus, and mild cerebellar tonsillar descent. The findings in (a) and (b) are consistent with intracranial hypotension.

On the fourth postoperative day, an urgent unenhanced CT scan of the head was performed after the patient developed altered mental status, confusion, disorientation, and slurred speech. The CT scan revealed areas of acute haemorrhage in both cerebellar hemispheres, with mass effect on the fourth ventricle and the brainstem and mild obstructive hydrocephalus (Figure 2). Subsequent review of nursing charts revealed excess CSF drainage over the previous night; thus, immediate clamping of the lumbar drain was performed, and an external ventricular drain (EVD) was placed by the neurosurgery team. MRI scanning of the brain, with and without contrast, revealed evidence of intracranial hypotension (Figure 3).

On the fourteenth postoperative day, the patient had an open surgical dural repair using direct suture closure, along with DuraGen® (a synthetic dural allograft), and fibrin glue. Additionally, the lumbar drain was successfully removed.

The patient's subsequent hospital course was complicated by deep venous thrombosis and respiratory failure, and he was ultimately discharged to the rehabilitation unit after EVD removal, ten days after the open dural repair surgery. No residual neurological deficits were present at the time of discharge.

## 2. Discussion

Remote cerebellar haemorrhage (RCH) refers to a postoperative parenchymal cerebellar haemorrhage, remote from the site of surgery. It may result as a consequence of cranial or spinal surgery, due to the opening of CSF cisterns, drainage of the ventricular system, or inadvertent tearing of the dura (dural tears having been reported in up-to 0.3% to 5.9% patients undergoing spinal surgery) [1]. Of note, while RCH is more commonly seen after supratentorial procedures, it may also occur following infratentorial or spinal surgeries.

Statistically, in 46% of cases, patients developed RCH within 10 hours of surgery; in 17% of cases, within 10 to 20 hours; in another 17% of cases, within 20 to 30 hours; and in 3% of cases, within 30-40 hours; and in the remaining 17% of cases, RCH developed more than 40 hours after surgery [2].* In our case, RCH was diagnosed four days after spinal surgery*.

Patients with RCH usually present predominantly with cerebellar signs ranging from mild nonspecific symptoms, such as headaches (with postural headaches being more specific for this disorder), dizziness, peduncular tremors, and vomiting. More aggressive signs such as motor deficits, gait ataxia, prolonged awakening from anesthesia, decreased levels of consciousness (with possible anxiety, agitation, or coma), neurological deterioration, or even death may occur, depending on the extent of the haemorrhage, and the severity of secondary effects [3–7]. But, of note, most cases are found incidentally on postoperative CT or MRI imaging.

While the precise mechanism of RCH is largely unknown, several theories have been proposed in recent years.

Sinking brain syndrome [8]: excess peri- or postoperative CSF drainage displaces the cerebellum inferiorly. Resultant stretching, occlusion, and tearing of the superior vermian vein and the superior cerebellar vein, which drain into the deep venous system, causes a venous infarct, whereas the overall increase in venous pressure ruptures the venous circulation, triggering a haemorrhage [9]. Bilateral cerebellar haemorrhage, as in our case, supports a venous etiology of the parenchymal haemorrhage.

Transient traction, tearing, kinking, or spasm of the superior cerebellar arteries [1, 3, 6, 7, 10–12]: a study by Mokri et al. demonstrated that brain sagging occurred in patients with spinal, but not cranial, CSF leaks [13]. Bloch et al. also reported similar results, as considerable clinical improvement occurred with external ventricular drainage and Trendelenburg or head-down positioning, via reversal of CSF flow dynamics between the cranial and spinal compartments [14].

High blood pressure increases the gradient between intravascular and CSF pressure: the resulting cerebellar sag transiently occludes the superior bridging veins within the posterior fossa, thus inducing haemorrhagic venous infarction of the cerebellar parenchyma [14–17].

Arterial origin of RCH: intermittent compression of the superior cerebellar artery results in a cerebellar infarct, with resultant haemorrhagic transformation [12, 17–20].

Removal of a supratentorial space occupying mass: supratentorial mass resection reduces the intracranial pressure, hence increasing the transmural pressure of veins or venules, developing an RCH [21].

Imaging of an RCH may reveal the ‘Zebra sign', a horizontal curvilinear configuration of haemorrhage between the cerebellar folia [3] (along the superior cerebellum, [19] where most draining veins are located) [3, 16]. This pattern of alternating curvilinear hyperdense (blood) and hypodense (cerebellar parenchyma) stripes resembles a zebra's coat, hence giving the zebra sign its name [19]. Slight variations in the distribution of blood may be observed; for instance, RCH may cause unilateral or bilateral (as in our case) parenchymal haemorrhage. [2]

The prognosis for RCH is good in the vast majority of cases, with more than half of the patients showing only mild residual neurological symptoms or complete recovery, while death occurs in approximately 10-15% [2]. According to one study, 15% of patients died as a result of complications due to RCH [2]. Poor outcome was correlated with an increase in severity of the RCH, higher patient age, longer time interval between the onset of symptoms and diagnosis [2], infarction secondary to the RCH [2], and extensive postoperative loss of CSF due to suction drainage [12, 17–20].

Management of RCH largely depends on the neurological status of the patient and the nature of the etiologic lesion. Usually, smaller haemorrhages without focal neurological deficits display milder symptoms. In these cases, conservative management is advised with bed rest, analgesics, and antiedema drugs (e.g., corticosteroids, such as dexamethasone). Follow-up monitoring is usually performed with serial imaging. [4]

On the other hand, larger haemorrhages with rapidly deteriorating neurological status result in an increased intracranial pressure with potential compression of the brainstem and hydrocephalus; hence, immediate surgery to evacuate the hematoma and to decompress the posterior fossa via expanded suboccipital craniotomy, duraplasty, and CSF diversion procedures is of essence [3–5]. In addition, there should be continuous blood pressure monitoring of these patients [2].

Health care personnel should remain vigilant about the predisposing factors which may contribute to RCH, such as previous brain atrophy (causing enlarged perivascular spaces, as blood vessel support is lost) [3], patient positioning during surgery (supine, prone or sitting) [6] possibly impairing venous drainage due to intraoperative head rotation which results in obstruction of the jugular vein at the first cervical vertebral transverse process, arterial hypertension, anticoagulation therapy (peri and preoperative) [5], coagulopathy, aneurysm, arteriovenous malformation, older age, preoperative aspirin administration [16], male sex, intraoperative use of esmolol [16], preoperative seizures [16], preoperative antibiotic use [16, 19], and tumors [3, 21–24]. In such cases, avoiding suction on the draining tube is key to decrease the risk for developing an RCH [20]. Subgaleal drains may also contribute to the phenomenon by causing stretching of the cerebellar veins (due to the negative upward pressure). Of note, rapid loss of CSF may occur during, and especially after the surgery, when the CSF drains have been placed. [2]

Patients with unexplained neurological symptoms or deterioration after spinal surgery complicated by CSF leakage should be evaluated carefully, and cranial imaging with CT or MRI should be promptly performed [3]. If no drain has been inserted, a CSF leak should be ruled out (or sealed, if detected), under the guidance of imaging (CT or MRI myelogram may also be useful to identify the site of the CSF leakage) [2]. If RCH is suspected, all drains (with suction or open to gravity) should be checked for increased drainage volumes, and, if necessary, should be clamped, until RCH is excluded using a CT or an MRI scan of the brain [2].

## 3. Conclusion

Dural tears acquired during surgery and postoperative CSF hypovolemia are the major factors contributing to the development of a RCH [2, 6, 10, 25] in spinal surgery. Etiologically, RCH is most likely venous in origin, demonstrated by the rapid intraoperative and/or postoperative CSF loss [4, 18–20, 26–30] causing mechanical (shearing of cerebellar bridging veins) or hemodynamic (increase in venous blood pressure) effects [2]. RCH should be considered as a potential source of unexplained neurological decline in patients after spinal surgery. Postural headache, a sign of intracranial hypotension, may be a harbinger of this phenomenon. Due to the possible serious sequelae associated with this condition, prompt diagnosis is required to optimise patient outcome [4]. Hence, in patients with this complication, further loss of CSF should be prevented by clamping or removing the CSF drainage tubes [31].
